# Clinical validation and literature review of robot-assisted cerebral angiography

**DOI:** 10.1186/s41016-026-00426-w

**Published:** 2026-01-30

**Authors:** Qi Liu, Siming Gui, Yang Zhao, Fei Wang, Chen Xu, Qiuju Cui, Youxiang Li, Yuanli Zhao

**Affiliations:** 1 Department of Neurosurgery, Peking Union Medical College Hospital, Beijing, China; 2 Department of Neurosurgery, Beijing Tiantan Hospital, Beijing, China; 3 Department of Nursing, Peking Union Medical College Hospital, Beijing, China

**Keywords:** Neurointervention, Surgical robot, Cerebral angiography, Radiation protection, Learning curve

## Abstract

**Background:**

To validate the clinical safety and efficacy of a domestically produced robotic-assisted system (YDHB-NS01) for cerebral angiography and to review the current status, advantages, and challenges of robot-assisted technology in cerebrovascular interventions.

**Methods:**

From May to October 2025, 25 consecutive patients who underwent robotic-assisted cerebral angiography and 25 consecutive patients who underwent manual cerebral angiography at our center were prospectively enrolled. The primary endpoints were technical success rate and clinical success rate. Secondary endpoints included procedure time, fluoroscopy time, radiation dose, contrast volume, total angiography room time, device performance evaluation, and complication rate. Additionally, a literature review was conducted to summarize the applications and developments of various robotic systems in neurointervention.

**Results:**

All 50 (25 in the robotic-assisted group and 25 in the manual group) procedures were successfully completed with a 100% technical success rate. There were no differences between the two groups in patients’ demographic data, fluoroscopy time, patient radiation dose, contrast agent dose, or total angiography room time (all *p* > 0.05). The robotic-assisted group had a shorter procedure time than the manual group (27 [15, 143] vs. 38 [21, 105], *p *= 0.005). A learning curve for the robotic-assisted system was observed. The robotic-assisted system operated stably without malfunctions. No procedure-related or device-related complications occurred.

**Conclusion:**

The preliminary clinical application demonstrates that the YDHB-NS01 robot-assisted system is feasible for diagnostic cerebral angiography and shows early indications of safety and comparable procedural performance to those of conventional manual methods. Given the small, single-center cohort and the exploratory nature of this study, larger multicenter controlled trials are required to confirm these findings.

**Supplementary Information:**

The online version contains supplementary material available at 10.1186/s41016-026-00426-w.

## Background

Digital subtraction angiography (DSA) remains the gold standard for diagnosing cerebrovascular diseases, such as intracranial aneurysms, arteriovenous malformations, and arterial stenosis [1]. However, this procedure requires operators to work under fluoroscopic guidance, leading to prolonged exposure to ionizing radiation. Despite protective measures like lead aprons, the cumulative radiation dose poses significant health risks to medical staff, including cataracts and malignancies [2, 3]. To address this issue, vascular interventional robotic systems have been developed, aiming to allow operators to perform procedures remotely from the radiation zone, thereby enhancing safety. Since the early twenty-first century, significant progress has been made in this field. In 2020, Professor Youxiang Li’s team from Beijing Tiantan Hospital successfully performed the first robot-assisted cerebral angiography in China, marking the entry of this technology into the clinical exploration stage in the country [4, 5]. This article also aims to systematically review the development, current status, and challenges of robotic systems in cerebral angiography, to prospect their future applications and offer real-world clinical data on the performance of the robot mentioned above, reflecting how it is used in routine practice outside of a controlled, pre-market setting, which may provide additional insights into its operational efficiency.

## Methods

### Clinical data

From May to August 2025, 25 consecutive patients undergoing robot-assisted cerebral angiography and 25 patients who underwent manual angiography performed consecutively by the same operator during the same period at Peking Union Medical College Hospital were enrolled. All patients provided written informed consent for the procedure and the robot-assisted operation. All patients’ demographic parameters were obtained from the electronic medical record system.

### Surgical equipment and materials

1. *Robotic system*: The Cerebrovascular Interventional Surgical Assist System (model: YDHB-NS01 Ver 2.0) consists of a master console and a slave manipulator unit, connected via a local area network.
2. *Vascular sheath*: 5F vascular sheath (brand: Cordis)
3. *Catheters and guidewires*: Primarily a 5F single-curve catheter (brand: Cordis)
4. *Imaging and angiography equipment*: DSA machine (GE) with iopamidol as the contrast agent

### Preoperative training for a robotic-assisted system

All robot-assisted angiography procedures in this study were performed by a single neurosurgeon with less than 3 years of experience independently performing manual diagnostic cerebral angiography. Prior to commencing the clinical series, the operator completed a training program for the YDHB-NS01 robotic-assisted system. The program comprised (1) didactic sessions covering system architecture, operation workflow, safety features, and emergency/manual override procedures and (2) hands-on bench training using vascular phantoms to practice catheter and guidewire manipulation under fluoroscopic simulation. This operator was required to demonstrate safe device setup, accurate remote catheter manipulation, correct remote contrast injection workflow, and the ability to perform immediate manual takeover before independent clinical use.

### Surgical technique

For patients in the robotic-assisted group, patients were placed in the supine position. After standard draping and disinfection, local anesthesia was administered to the right groin. The 5F sheath was inserted into the femoral or radial artery using the Seldinger technique. The 5F single-curve catheter was then fixed into the propulsion system of the slave robot, manually introduced into the arterial sheath, and secured by the robotic arm. A three-way stopcock was connected to the pressure infusion system and to a heparinized saline drip. The operator, stationed in a control room outside the interventional suite, manipulated the master console. During robot-assisted procedures, the contrast was delivered by a remote-controlled power injector that was integrated with the robotic workflow and operated from the master console. The operator at the console triggered the injection under real-time fluoroscopic guidance. Under image guidance, commands were transmitted via the network to control the slave robot in the operating room. Real-time catheter movement feedback was transmitted to the master console, allowing the operator to perform the procedure, including roadmap acquisition and selective angiography as needed, ultimately completing robot-assisted bilateral carotid and vertebral angiography. For patients in the control group, the operator completed the cerebral angiography procedure manually, as usual.

### Observation indicators

1. *Technical success rate*: Successful catheter positioning and contrast opacification of the target vessels
2. *Clinical success rate*: Successful completion of cerebral angiography using the robotic-assisted system
3. *Procedure parameters*: Procedure time (from catheter entry to withdrawal), fluoroscopy time, total angiography room time, patient radiation dose (recorded by the DSA machine), and contrast agent volume
4. *Device performance evaluation*: Subjective assessment of console stability, catheter/guidewire delivery smoothness, manipulator flexibility, control handle responsiveness, and device failures (using an operator satisfaction survey, rate from 0 to 10 scores).
5. *Complications*: Detailed recording of any perioperative complications (until discharge), including puncture-related (hematoma, spasm, dissection), operation-related (vessel spasm, dissection, perforation, embolism), neurological complications (TIA, stroke), and contrast-related reactions (allergy, nephropathy).

### Statistical analysis

Continuous variables were tested for normality using the Shapiro–Wilk test. Normally distributed continuous variables are presented as mean ± standard deviation (SD); non-normally distributed variables are presented as median (range). Group comparisons (robot vs. control/manual) were performed with Student’s *t*-test for normally distributed variables and the Mann–Whitney *U*-test for non-normal variables. Categorical variables were compared using the *χ*^2^ test or Fisher’s exact test as appropriate. All tests were two-sided, and a *p*-value < 0.05 was considered statistically significant. We report 95% confidence intervals (CIs) for key estimates. Analyses were performed using SPSS statistical software.

## Results

### Procedure completion

All 50 patients (25 with manual angiography and 25 with robotic-assisted angiography) successfully underwent cerebral angiography, resulting in a 100% clinical and technical success rate. All target vessels were clearly visualized, meeting diagnostic requirements.

### Procedure parameters

**Fig. 1:**
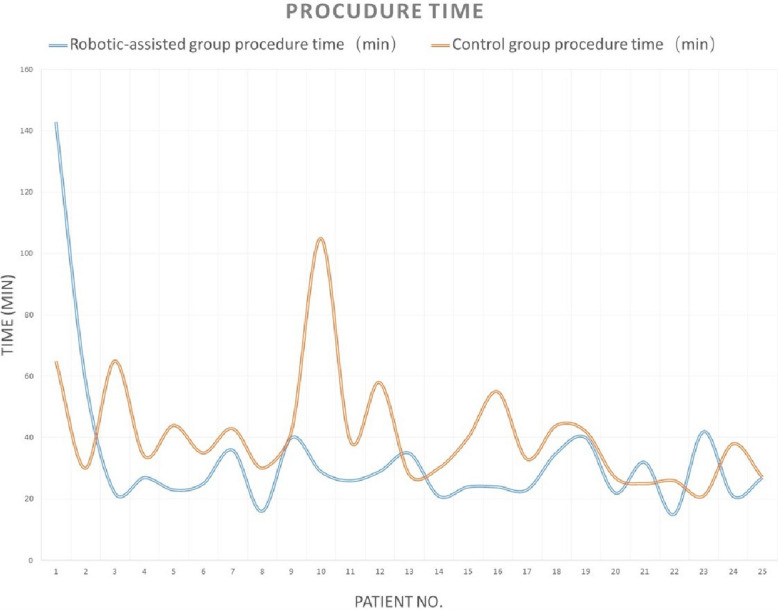
Learning curve of procedure time for robotic-assisted cerebral angiography compared to manual cerebral angiography

**Fig. 2:**
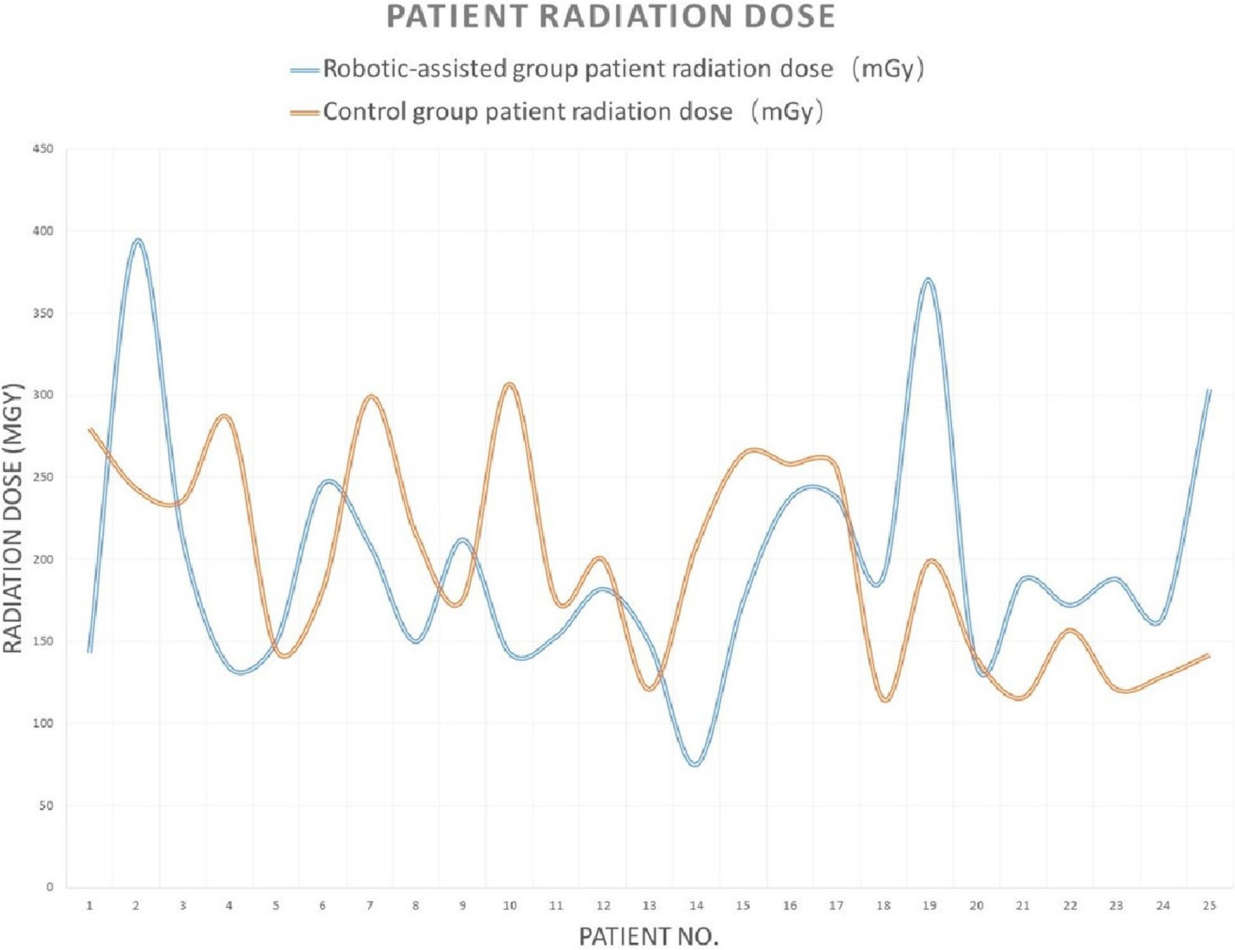
Learning curve of patient radiation dose for robotic-assisted cerebral angiography compared to manual cerebral angiography

**Fig. 3:**
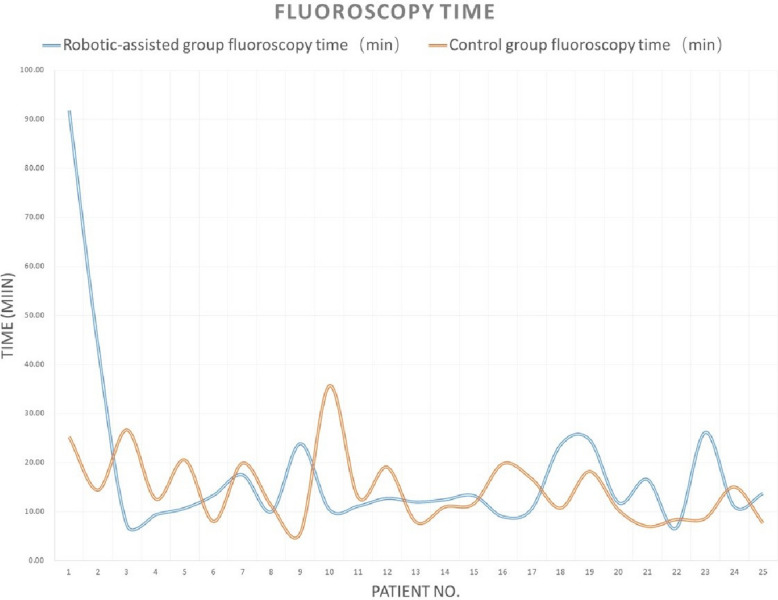
Learning curve of fluoroscopy time for robotic-assisted cerebral angiography compared with manual cerebral angiography

**Fig. 4:**
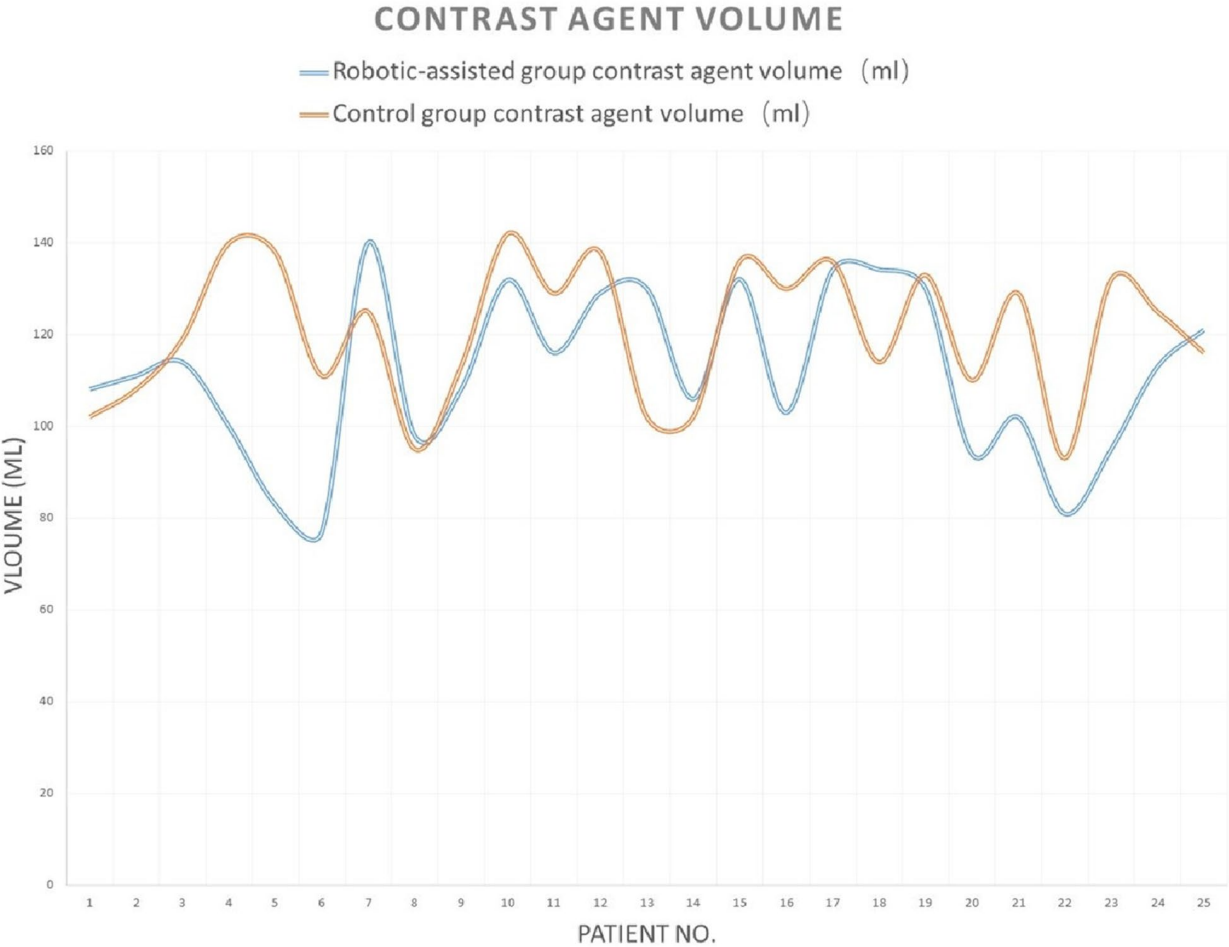
Learning curve of contrast agent volume for robotic-assisted cerebral angiography compared with manual cerebral angiography

There were no differences between the two groups in patients’ demographic data (age, gender, hypertension, and diabetes), fluoroscopy time, patient radiation dose, contrast agent dose, or total angiography room time (all *p* > 0.05). The robotic-assisted group had a shorter procedure time than the manual (control) group (27 [15, 143] vs. 38 [21, 105], *p* = 0.005) (Table 1). Tables 2 and 3 provided the detailed data for 50 patients. Analysis revealed a significant learning curve for the robotic-assisted group: the first 2 cases required longer times and a higher radiation dose, and the subsequent 23 cases showed more stabilized parameters in procedure time, patient radiation dose, fluoroscopy time, and contrast agent volume (Figs. 1, 2, 3, 4).

**Table 1:** Demographic and procedural parameters comparison between the robotic-assisted angiography group and the manual angiography (control) group

	Robotic-assisted group (*N* = 25)	Control group (*N* = 25)	*p*-value
Male	12 (48%)	11 (44%)	0.777
Age	60 [41, 75]	60 [38, 74]	0.602
Hypertension	14 (56%)	10 (40%)	0.258
Diabetes	6 (24%)	3 (12%)	0.269
Procedure time	27 [15, 143]	38 [21, 105]	**0.005**
Patient radiation dose	182 [75, 394]	199 [115, 307]	0.901
Fluoroscopy time	12.40 [6.63, 91.75]	12.57 [5.67, 35.65]	0.719
Contrast agent volume	111 [77, 140]	125 [93, 142]	0.084
Total angiography room time	47 [31, 170]	48 [36, 123]	0.676

**Table 2:** Demographic parameters between the robotic-assisted angiography group and the manual angiography (control) group

	Gender		Age		Hypertension (*Y* = 1, *N* = 0)		Diabetes (*Y* = 1, *N* = 0)		Diagnosis	
	Robotic-assisted group	Control group	Robotic-assisted group	Control group	Robotic-assisted group	Control group	Robotic-assisted group	Control group	Robotic-assisted group	Control group
1	Male	Male	49	40	1	0	0	0	Intracranial aneurysm	bAVM
2	Female	Female	69	63	1	0	0	0	dAVF	Intracranial aneurysm
3	Female	Female	41	69	0	1	0	0	Intracranial aneurysm	ICAS
4	Male	Male	69	66	1	0	1	0	ICAS	Intracranial aneurysm
5	Female	Female	44	68	0	0	1	0	ICAS	Intracranial aneurysm
6	Female	Female	58	62	1	1	1	0	Intracranial aneurysm	Intracranial aneurysm
7	Female	Male	75	49	1	1	0	0	Intracranial aneurysm	Intracranial aneurysm
8	Male	Male	51	52	1	1	0	0	ICAS	ICAS
9	Female	Female	74	54	1	0	0	0	ICAS	Intracranial aneurysm
10	Male	Female	65	61	1	0	0	0	Intracranial aneurysm	dAVF
11	Female	Male	41	60	0	1	0	0	Intracranial aneurysm	ICAS
12	Female	Female	71	57	1	1	1	0	Intracranial aneurysm	Intracranial aneurysm
13	Male	Male	54	66	0	1	0	0	Moyamoya disease	ICAS
14	Female	Female	55	38	1	0	0	0	Intracranial aneurysm	ICAS
15	Female	Female	70	57	0	0	0	0	Intracranial aneurysm	dAVF
16	Male	Female	47	58	0	0	0	0	Intracranial aneurysm	Intracranial aneurysm
17	Female	Male	74	62	1	0	1	0	Intracranial aneurysm	Intracranial aneurysm
18	Female	Female	61	49	1	0	0	0	Intracranial aneurysm	Intracranial aneurysm
19	Male	Male	51	62	0	0	0	1	Moyamoya disease	ICAS
20	Male	Male	60	51	1	0	0	1	Intracranial aneurysm	ICAS
21	Male	Female	72	74	1	1	1	0	ICAS	Intracranial aneurysm
22	Male	Female	50	43	0	1	0	1	Moyamoya disease	ICAS
23	Male	Male	71	51	0	1	0	0	ICAS	Intracranial aneurysm
24	Female	Female	57	65	0	0	0	0	Moyamoya disease	Intracranial aneurysm
25	Male	Male	61	70	0	0	0	0	ICAS	Intracranial aneurysm

*bAVM* brain arteriovenous malformation, *dAVF* dural arteriovenous fistula, *ICAS* intracranial arterial stenosis

**Table 3:** Procedure parameters between the robotic-assisted angiography group and the manual angiography (control) group

	Procedure time (min)		Patient radiation dose (mGy)		Fluoroscopy time (min)		Contrast agent volume (ml)		Total angiography room time (min)		Operator satisfaction rate for robotic-assisted system
	Robotic-assisted group	Control group	Robotic-assisted group	Control group	Robotic-assisted group	Control group	Robotic-assisted group	Control group	Robotic-assisted group	Control group	
1	143	65	143	280	91.75	25.23	108.1	102	170	75	7
2	59	30	394	243	43.53	14.42	111	108	78	39	6
3	22	65	212	236	7.20	26.67	114	119	38	80	8
4	27	34	134	285	9.37	12.57	100	140	47	45	9
5	23	44	150	145	10.65	20.55	83	138	46	53	9
6	25	35	246	182	13.32	8.02	77	111	48	48	10
7	36	43	209	299	17.48	19.92	140	125	61	51	9
8	16	30	150	215	9.97	11.17	98	95	31	42	8
9	40	42	212	176	23.83	5.67	108	113	59	56	9
10	29	105	143	307	10.40	35.65	131.8	142	47	123	9
11	26	39	153	175	11.10	12.90	116	129	39	48	10
12	29	58	182	200	12.68	19.07	129	138	49	72	10
13	35	28	149	121	11.93	7.88	130	102	53	40	10
14	21	30	75	208	12.40	11.00	106	102	46	48	9
15	24	40	174	264	13.23	11.63	132	136	46	49	8
16	24	55	237	258	8.93	19.78	103	130	45	69	9
17	23	33	238	256	10.53	16.72	134	136	43	47	7
18	35	44	189	115	23.60	10.73	134.1	114	54	59	7
19	40	42	370	199	24.65	18.20	130	133	65	57	8
20	22	27	136	140	11.80	10.38	94	110	41	41	9
21	32	25	188	116	16.53	7.00	102	129	50	38	8
22	15	26	172	157	6.63	8.43	81	93	41	39	10
23	42	21	188	121	26.12	8.65	95	132	69	36	10
24	21	38	165	129	11.05	15.05	113	125	39	56	8
25	27	27	304	142	13.67	7.70	121	116	41	37	8

### Device performance

The robotic system operated stably throughout all procedures without any mechanical or system failures. Operators reported smooth catheter and guidewire delivery, firm and flexible manipulator fixation, responsive control handles, and good force feedback, enabling precise catheter manipulation.

### Complications

No complications occurred in any of the 50 patients. Specifically, there were no puncture-related complications (e.g., hematoma, dissection, pseudoaneurysm), no operation-related complications (e.g., vessel spasm, injury, perforation, thromboembolism), no neurological complications (e.g., TIA, seizure, ischemic stroke), and no contrast-related adverse reactions (e.g., allergy, nausea, vomiting, nephropathy).

## Discussion

This is a single-center post-marketing case series. Our findings demonstrate that robotic-assisted angiography achieves comparable outcomes (no differences in fluoroscopy time, patient radiation dose, contrast agent dose) to manual angiography while reducing procedure time, and that no complications occurred. We believe the reduction in procedure time in the robotic-assisted group stems from several factors. First, this robotic-assisted system incorporates a specialized one-way valve mechanism that maintains continuous high-pressure heparin saline infusion or contrast administration without three-way stopcock switching. Second, the robot incorporates a dedicated guidewire holder, eliminating the need for repeated insertion of the guidewire into and removal from the catheter during the procedure. Third, with the control console (including fluoroscopy, road map, angiography, operating table and C-arm movement) positioned outside the operating room, surgeons avoid frequent entry into the operating room, allowing the entire procedure to proceed with fluidity. We believe these factors likely contribute to the shorter operating times observed in the robotic group compared to the conventional group. However, there was no difference in total operative time between the two groups. We believe this may be due to the longer preoperative set-up time required for the robotic-assisted group compared to the manual group.

Besides, we observed a clear early learning effect: the first two procedures had substantially longer procedural and fluoroscopy times. These two procedures were performed by the operator after the training period. We believe the operator had to undergo real-world familiarization with device setup and console workflow. Second, the second patient had a dural arteriovenous fistula requiring multiple extra- and internal carotid artery injections and had limited cooperation under local anesthesia, which necessitated repeated angiographic acquisitions. The subsequent 23 procedures were stabilized with shorter and more consistent procedural and fluoroscopy times. Because all robot-assisted procedures were performed by one relatively inexperienced operator, the early learning curve likely reflects individual adaptation to the robotic workflow. Centers with multiple operators may experience slightly different early-implementation patterns.

In this single-center, post-market series of 25 consecutive diagnostic cerebral angiographies, the YDHB-NS01 robot-assisted system proved feasible to use and demonstrated early signals of safety. The learning curve observed in this series likely reflects predictable human-factor and workflow adaptations required when introducing a new robotic platform into routine clinical practice. However, these results should be interpreted with caution because of the small sample size, single-center design, limited follow-up, and the exploratory nature of the study. Future research should include larger, prospective, multicenter studies with randomized or matched control groups, standardized operator-experience criteria, and cost-effectiveness analyses to fully evaluate the safety, efficacy, and clinical utility of robotic assistance in cerebral angiography and neurointervention.

## Literature review

### Introduction

Endovascular interventions have become preferred treatments for cerebrovascular diseases due to minimal trauma and rapid recovery [1–3]. However, operator’s exposure to radiation and physical strain from lead aprons are significant drawbacks [2, 3]. Robot-assisted technology offers a solution and has advanced in various clinical fields [4, 5]. Cerebrovascular anatomy’s complexity makes interventions challenging, leading to slower robotic adoption. Nevertheless, specialized systems are now used in cerebral angiography, carotid stenting, and aneurysm treatment [6–9].

### Robot-assisted systems in clinical use

Early systems focused on linear movements; modern systems allow translational and rotational control, typically using a master–slave architecture [5]. This setup enhances operator safety and comfort. Current neurointerventional systems include the CorPath GRX [10], Magellan [11], VIR-2 [12], PANVIS-A [13], and YDHB-NS01 [14]. We conducted a systematic search of PubMed/MEDLINE, Embase, and Web of Science for studies published up to September 30, 2025, which evaluated robotic assistance in cerebral or neurovascular intervention. The search strategy combined terms for robotics and neurointervention (e.g., “robot,” “robotic,” “robot-assisted,” “neurointerv,” “neuro-vascular,” “angiograph,” “endovascular,” “cerebral,” “intracranial,” and specific device names such as “CorPath,” “Magellan,” “VIR-2,” “PANVIS-A,” “YDHB-NS01”). Inclusion criteria were as follows: (1) Original clinical studies (randomized trials, prospective or retrospective cohorts, case series > 5 patients) reporting outcomes of robotic-assisted cerebral angiography or neuroendovascular procedures and (2) reporting at least one procedural outcome (technical success, fluoroscopy time, radiation dose, complications). We excluded animal/laboratory-only studies, reviews, and editorials. We summarized the clinical performance of various robotic-assisted systems as reported in the previous literature below. Supplemental Table 1 summarizes the clinical studies for the above robotic-assisted systems used in cerebral angiography and other neurointerventional procedures.

1. *CorPath GRX system*

Primarily used for cardiovascular interventions, modified versions are applied in neurovascular cases [15–17]. It offers precise guidewire/catheter control and catheter support. A prospective multicenter study of 117 aneurysm patients reported a 94% technical success rate [7]. Complete occlusion (Raymond-Roy grade 1) was achieved in 64.5% of cases. Complication rates were low (3.4% intraoperative). Limitations include inability to manipulate two catheters simultaneously, lack of haptic feedback, longer procedure times, and higher costs [7, 9, 18, 19]. Future improvements should focus on multi-catheter control and enhanced feedback.

(2) *Magellan system*

Initially used for peripheral vascular procedures, it has been used in cerebral angiography [20, 21]. A study of nine angiographies found no significant differences in procedure time, fluoroscopy time, or contrast dose compared with manual methods, with no complications [21]. It reduces vessel wall impact, minimizing endothelial damage [11].

(3) *VIR-2 system*

This system includes a master–slave setup and a 3D imaging system for enhanced navigation [12]. A study of 15 patients reported a mean procedure time of 34.4 ± 5.13 min, with operators remaining radiation-free. All steps post-puncture were robot-assisted, with no complications [12]. Limitations include inconvenient manipulation and console layout inefficiencies.

(4) *YDHB-NS01 system*

This domestically developed Chinese system was validated in a prospective, multicenter RCT involving 260 patients [14]. Angiography success rates were 100% in both the robotic and the manual groups, with no significant differences in procedure time or patient radiation dose. Safety profiles were comparable, featuring minor complications, such as a puncture site hematoma. This study confirmed its non-inferiority to conventional angiography, paving the way for complex procedures such as aneurysm embolization. Our study’s results corroborate these findings, showing high success rates and a manageable learning curve.

(5) *PANVIS-A system*

It features an intuitive fingertip control system (COF) and supports multi-instrument coordination [13]. An RCT involving 128 patients demonstrated a 96% reduction in operator radiation dose in the robotic group compared to the manual group, with no difference in procedure success or time, albeit with longer setup times [22].

### Advantages and limitations

Robotic systems offer submillimeter precision, reducing operator fatigue and errors [13, 14, 23]. What’s more, previous studies have associated spinal disorders with annual procedural volume and years of clinical practice, areas in which robotic assistance can be beneficial by alleviating physical demands and mitigating operator fatigue [24]. Additionally, interventionalists can operate the procedure remotely while seated comfortably, eliminating the need for lead aprons and minimizing back discomfort and the risk of orthopedic injuries [25]. However, key limitations persist: lack of haptic feedback, incomplete automation (e.g., manual access), limited operational length for distal vessels, and low intelligence levels. Our experience confirms the need for haptic feedback development.

### Challenges and prospects

Challenges include perfecting force feedback, optimizing image fusion accuracy, and improving compatibility with commercial devices to reduce costs [23]. Future directions involve integrating AI for image registration and developing universal platforms. Ultimately, robotics may encompass the entire “diagnosis-treatment-prognosis” continuum, improving precision and resource allocation. The promising results from our center and others suggest an expanding role for robotics in neurointervention.

## Supplementary Information

Additional file 1: Supplemental Table 1 Summary of clinical researches for robotic-assisted system used in cerebral angiography/neurointerventional procedures.

## Data Availability

No datasets were generated or analysed during the current study.

## Abbreviations

DSA: Digital subtraction angiography
SD: Standard deviation
CIs: Confidence intervals
COF: Control system
