# Supplementary material for: Clinical validation and literature review of robot-assisted cerebral angiography
Source: Chin Neurosurg J. 2026 Jan 30;12:4. doi: 10.1186/s41016-026-00426-w (PMC12857158; doi:10.1186/s41016-026-00426-w)
Supplement: Supplementary file 1 — Additional file 1: Supplemental Table 1 Summary of clinical researches for robotic-assisted system used in cerebral angiography/neurointerventional procedures. [file 41016_2026_426_MOESM1_ESM.docx]

Supplemental Table 1 Summary of clinical researches for robotic-assisted system used in cerebral angiography/neurointerventional procedures

|  | Study | Year | Country | Robotic-assisted System | Study Design | N | Procedure types | Procedural outcomes | Complication Rate | Limitation of study |
| --- | --- | --- | --- | --- | --- | --- | --- | --- | --- | --- |
| 1 | Cancelliere et al | 2022 | Canada | CorPath GRX | Case series | 6 | Intracranial aneurysm embolization | The technical success rate of the procedures was 100% | 0 | Not mentioned |
| 2 | Mendes Pereira et al | 2022 | Canada | CorPath GRX | Case series | 6 | Intracranial aneurysm embolization | All clinical procedures were technically successful | 0 | A single-center pilot study |
| 3 | Chivot et al | 2024 | France | CorPath GRX | Case series | 10 | Intracranial aneurysm embolization | Only one partial conversion to a manual technique | 0 | Larger, in-depth studies are now warranted |
| 4 | Sajja et al | 2020 | USA | CorPath GRX | Case series | 10 | Carotid Artery Stenting and cerebral angiography | Conversion to manual control occurred in three cases | 0 | Not mentioned |
| 5 | Desai et al. | 2021 | USA | CorPath GRX | Case series | 6 | Cerebral angiography and dAVF embolization | 100% technical success | 0 | Not mentioned |
| 6 | Weinberg et al | 2020 | USA | CorPath GRX | Case series | 6 | Carotid Artery Stenting | technical success was achieved in all 6 (100%) procedures | 0 | Future clinical investigations among larger cohorts are needed to demonstrate reliable performance and patient benefit. |
| 7 | Abbas et al | 2022 | USA | CorPath GRX | Case series | 14 | Carotid Artery Stenting | All procedures were conducted successfully | 0 | Retrospective design, lack of randomization, lack of long-term patient follow-up, lack of objective criteria in choosing the robotic technique, small sample size |
| 8 | Costa et al | 2023 | USA | CorPath GRX | Retrospective Cohort | 41 | Carotid Artery Stenting and cerebral angiography | Procedural success was 83% and conversion from robotic-assisted to manual control was 17% | 0 | A retrospective design, lack of long-term patient follow-up, a relatively small sample size, a single-center study |
| 9 | Beaman et al | 2024 | USA | CorPath GRX | Retrospective case series | 113 | Cerebral angiography | 77.9% cases were completed successfully without unplanned manual conversion | 0 | Patients were  not chosen at random; did not make comparisons to manual operation |
| 10 | Mendes Pereira et al | 2024 | Canada | CorPath GRX | Prospective cohort | 117 | Intracranial aneurysm embolization | Primary effectiveness was achieved in 94% subjects | 3.4% | Not mentioned |
| 11 | Jones et al | 2021 | UK | Magellan | Case series | 13 | Carotid Artery Stenting | Technical success was achieved in all of patients | 0 | A small and non-comparative series |
| 12 | Liu et al | 2024 | China | 1^st^ Generation YDHB-NS01 | RCT | 128 | Cerebral angiography | The success rate of angiography was 100%. The rate of the catheter reaching the target vessel was 100.00% | 0 | Not mentioned |
| 13 | Zhang et al | 2025 | China | PANVIS-A | RCT | 64 | Cerebral angiography | 100% clinical success and a 100% technical success | 0 | Underpowered sample size; patients with planned simultaneous therapeutic procedures during angiography were excluded from this study |
